# Renal safety of intraoperative local vancomycin powder application in orthopedic surgery: a retrospective analysis

**DOI:** 10.5194/jbji-10-609-2025

**Published:** 2025-12-19

**Authors:** Juliane Beschauner, Maria Felsberg, Alexander Zeh, Karl-Stefan Delank, Natalia Gutteck, Felix Werneburg

**Affiliations:** 1 Department of Orthopedic and Trauma Surgery, Martin Luther University Halle-Wittenberg, Halle, Germany

## Abstract

**Introduction**: Topical vancomycin powder is increasingly used in orthopedic surgery to prevent surgical site infections (SSIs). While its efficacy is well established, data on systemic safety – particularly renal effects – are limited. Given vancomycin's known nephrotoxicity when administered systemically, we evaluated whether local intraoperative application affects short-term renal function. **Methods:** This retrospective single-center cohort included 50 adults who underwent orthopedic surgery with the application of intraoperative topical vancomycin powder (January–July 2024). Serum creatinine (SCr) and estimated glomerular filtration rate (eGFR) were measured preoperatively and at two routine postoperative time points. The primary endpoint was acute kidney injury (AKI) per KDIGO serum creatinine criteria; secondary endpoints were within-patient changes in SCr and eGFR. Prespecified subgroups were nephrotoxic concomitant medication (yes/no), application site (epifascial/subfascial), vancomycin dose ( < 1000 vs. ≥ 1000 mg), and indication (aseptic/septic). Analyses used Wilcoxon signed-rank and Fisher's exact tests. **Results**: The most common applied dose was 1000 mg (48 %; median 1000 mg; range 500–4000). Postoperative labs were obtained at median day 1 and day 3. AKI occurred in 2/50 patients (4 %), both stage 1; no stage 2–3 events were observed. Both AKI cases had concomitant exposure to potentially nephrotoxic medication; however, AKI incidence did not statistically differ across prespecified subgroups (nephrotoxic co-medication, application plane, dose category, indication; all p>0.05 ). Paired analyses showed minimal within-patient change in renal indices: eGFR exhibited no central shift from baseline at either time point, and serum creatinine showed no systematic postoperative increase. **Conclusions**: Local intraoperative vancomycin powder application was not associated with short-term renal impairment. These findings support its renal safety in orthopedic surgery. Prospective trials with pharmacokinetic monitoring are warranted to confirm long-term safety. *Level of evidence*: IV (retrospective case series).

## 1 Introduction

Surgical site infections (SSIs) are serious complications in orthopedic surgery, with incidence rates reported between < 1 % and 20 % depending on the procedure and patient risk factors (Berríos-Torres et al., 2017). Such infections lead to increased morbidity, prolonged hospitalization, and higher healthcare costs (Berríos-Torres et al., 2017). Standard prophylaxis with intravenous antibiotics reduces SSI risk, but breakthroughs still occur, especially against resistant organisms (Bratzler et al., 2013; Hawn et al., 2013). This has prompted interest in adjunct local antibiotic measures. A widely adopted strategy is the intraoperative application of vancomycin powder directly into the surgical wound. Multiple studies and meta-analyses have demonstrated a significant reduction in SSIs with this technique, while emphasizing its ease of use, safety, and cost-efficiency (Chiang et al., 2014). Vancomycin is attractive for this purpose due to its convenient powder form and potent activity against Gram-positive bacteria, including methicillin-resistant *Staphylococcus aureus* (MRSA), which are common causes of orthopedic SSIs (Matziolis et al., 2020). Originally adopted in cardiothoracic surgery to prevent sternal wound infections, topical vancomycin prophylaxis was later popularized in spine surgery and is now employed across multiple orthopedic subspecialties (Martinez-Peralta et al., 2022). In spinal surgery, intrawound vancomycin has been shown to markedly lower infection rates, without contributing to a long-term increase in vancomycin-resistant organisms (Shu et al., 2023; Khanna et al., 2019). Likewise, in joint arthroplasty – particularly during septic revision procedures – local vancomycin powder is increasingly employed as part of the possible antimicrobial regimen (Bunea et al., 2024).

Numerous studies have evaluated the clinical efficacy of intrawound vancomycin for SSI prevention. A large multicenter randomized trial in high-risk tibial plateau and pilon fractures ( n=980 ) found that adding 1 g of vancomycin powder at wound closure reduced the 6-month deep SSI rate from 9.8 % to 6.4 % (Higgins et al., 2023). While this did not reach overall statistical significance ( p=0.06 ), post hoc analysis confirmed a significant decrease in Gram-positive deep infections ( p=0.02 ). In spinal surgery, Zhang et al. (2021) observed a 90 d SSI incidence of only 1.3 % with 1 g of intrawound vancomycin versus 10.3 % in the control group – a relative reduction of 87.5 % ( p<0.05 ) (Zhang et al., 2021). In arthroplasty, a 2024 meta-analysis including 24 studies and over 34 000 patients showed a consistent benefit in reducing SSIs and periprosthetic joint infections (PJIs) across joint types and surgical settings (Chan et al., 2024). Nevertheless, the broader literature is not uniform; several higher-quality evaluations (including randomized trials and systematic reviews) have found mixed or null effects, so overall efficacy remains debated (Mancino et al., 2023).

Despite its widespread use, comprehensive data on renal outcomes following local vancomycin application in orthopedic surgery remain limited. Most studies focus primarily on infection prevention efficacy, with few systematically evaluating renal safety parameters. However, intravenous administration of vancomycin is associated with a well-documented risk of nephrotoxicity. The incidence of vancomycin-associated acute kidney injury (VA-AKI) varies widely, with reported rates ranging from 5 % to 43 %, depending on patient populations, dosing regimens, and concomitant nephrotoxic agents (Kyaw et al., 2025). Therefore, equally important is the safety profile of local vancomycin, particularly regarding renal toxicity. Vancomycin's known nephrotoxic potential raises concern that topical application may lead to systemic uptake and acute kidney injury (AKI). However, recent studies have shown minimal systemic absorption. In a prospective study of patients undergoing thoracolumbar spinal fusion, Sweet et al. (2011) found serum vancomycin concentrations to be undetectable in most cases; when present, levels remained well below the nephrotoxic threshold (Sweet et al., 2011). In a prospective study, O'Toole et al. (2022) reported undetectable serum vancomycin levels in all but two patients after 1 g topical application, and no clinically relevant creatinine increases were observed (O'Toole et al., 2022).

Finally, there is no consensus on optimal vancomycin dosing. In this context, Brothers et al. (2023) have highlighted that sub-inhibitory dosing of vancomycin may paradoxically increase the risk of surgical site infections through enhanced biofilm formation, underscoring the importance of appropriate local dosing strategies (Brothers et al., 2023). Most studies apply 1–2 g per procedure. One trial comparing 1 to 2 g in spine fusion reported similar infection rates (Lee et al., 2020). Meta-analyses also confirm efficacy across this dosing range (Chan et al., 2024). Notably, higher doses have been associated with increased sterile wound complications, such as delayed healing and serous drainage (Chan et al., 2024).

In summary, intraoperative vancomycin powder is a promising adjunct to reduce SSIs in orthopedic surgery. It offers high local concentrations with minimal systemic toxicity and low resistance risk. However, uncertainty remains regarding optimal dosing, systemic effects, and specific patient populations most likely to benefit. The present study aims to specifically address whether the intraoperative application of vancomycin powder is associated with measurable changes in renal function, such as variations in serum creatinine (SCr) and estimated glomerular filtration rate (eGFR), in a mixed orthopedic population. By systematically analyzing renal parameters before and after surgery and by considering potential influencing factors such as vancomycin dosage, application site, and concurrent nephrotoxic medication, this study seeks to provide evidence-based data on the systemic safety of this increasingly common approach to infection prevention and adjunctive treatment in orthopedic surgery.

## 2 Methods

This retrospective observational study was conducted at the Department of Orthopedics, Trauma and Reconstructive Surgery at Martin Luther University Halle-Wittenberg. It included patients who underwent orthopedic surgery between January and July 2024 and received intraoperative local application of vancomycin powder.

Eligible cases involved adult patients undergoing elective or urgent orthopedic procedures, including spinal surgery, joint arthroplasty, and revision procedures, in which vancomycin powder was applied locally during the operation. The powder was administered either epifascially (above the fascia) or subfascially (directly into the wound bed), as documented in the operative reports. Data were extracted retrospectively from electronic medical records.

The following variables were collected:

– Demographic information: age and sex
– Surgical characteristics: procedure type, vancomycin dose (in milligrams), route of application (epifascial or subfascial)
– Concomitant medication: use of potentially nephrotoxic agents
– Laboratory parameters:
  – Preoperative/Postoperative: serum creatinine ( µ mol L^−1^) and estimated glomerular filtration rate (eGFR, mL/min/1.73 m^2^)
  – Note: All eGFR values were converted into numeric equivalents for statistical analysis (e.g., “ >90 ” was interpreted as 90 mL/min/1.73 m^2^).

Patients with incomplete documentation of the relevant laboratory parameters were excluded from the analysis.

### 2.1 Timing of laboratory assessments

Per institutional routine, postoperative assessments are scheduled for postoperative days 1 and 3 and were prespecified as target time points. Because clinical logistics may shift sampling, actual timing was calculated as days from the surgery date/time to the lab collection timestamp in the EHR; these values are reported descriptively.

### 2.2 Exposure definitions – nephrotoxic agents

Prespecified potentially nephrotoxic exposures included systemic vancomycin, aminoglycosides (gentamicin, tobramycin, amikacin), piperacillin/tazobactam, ampicillin/sulbactam, antistaphylococcal penicillins (flucloxacillin/oxacillin), non-steroidal anti-inflammatory drugs (ibuprofen, diclofenac, etoricoxib), and iodinated contrast media. Low-molecular-weight heparins (enoxaparin, tinzaparin) were recorded as renally cleared agents but were not classified as “nephrotoxic” for subgroup analyses; dosing of renally cleared drugs followed guideline-based protocols and was adjusted to kidney function when indicated.

### 2.3 Outcomes

The clinically relevant primary safety endpoint was acute kidney injury (AKI) defined by KDIGO serum creatinine criteria. As supportive measures of renal function, we summarized within-patient changes in serum creatinine and eGFR between baseline and each postoperative time point.

### 2.4 Statistical analysis

Analyses were performed in IBM SPSS Statistics v27 (IBM Corp.). Continuous variables are presented as mean ± SD or median (IQR), and categorical variables are presented as counts and percentages. Within-patient changes in SCr/eGFR were analyzed using the Wilcoxon signed-rank test and summarized descriptively. The subgroup analysis evaluated whether AKI incidence differed by nephrotoxic concomitant medication (yes vs. no), application plane (epifascial vs. subfascial), vancomycin dose ( <1000 vs. ≥1000 mg), and surgical indication (aseptic vs. septic) using Fisher's exact tests. Two-sided p<0.05 was considered statistically significant.

## 3 Results

A total of 50 patients were included in this retrospective analysis. The cohort comprised 29 males (58 %) and 21 females (42 %). The mean age was 64.2 years (SD ± 20.5), with a median of 69.5 years. Patient ages ranged from 16 to 93 years. Of the 50 patients included, 31 procedures (62 %) were performed for aseptic indications, while 19 cases (38 %) involved surgery for septic conditions, such as periprosthetic joint infections or soft tissue infections. The plane of local vancomycin application was clearly documented in 33 out of 50 patients (66 %): epifascial in 19/50 (38 %) and subfascial in 14/50 (28 %).

### 3.1 Vancomycin dosage

**Figure 1:**
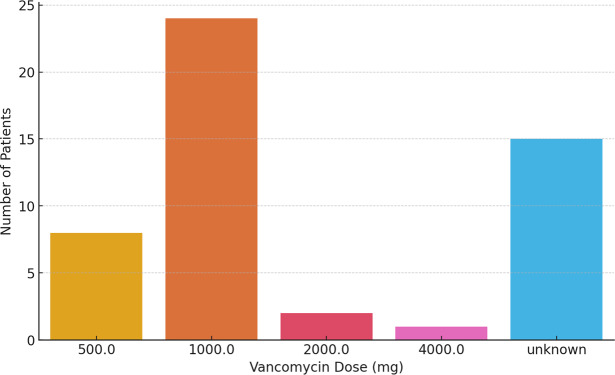
Frequency distribution of intraoperatively administered vancomycin doses.

Vancomycin dosage was documented in 35 out of 50 cases (70 %). Among these, the administered dose ranged from 500 to 4000 mg. The median dose was 1000 mg, and the mean dose was 1028 mg (SD ± 618 mg). The most frequently used dose was 1000 mg, which was administered in 24 patients (48 %). In 30 % of patients, no specific dosage information was available from the records. The distribution of administered vancomycin doses, including undocumented cases, is shown in Fig. 1.

Vancomycin powder is normally dosed in 500 or 1000 mg increments. In our cohort, dosing varied slightly by application plane. Among patients with both site and dose documented, epifascial cases ( n=16 ) received a median of 1000 mg (range 500–2000; mean 937.5 ± 359.4 mg), and subfascial cases ( n=14 ) received a median of 1000 mg (range 500–2000; mean 1035.7 ± 307.9 mg). The sample for this analysis is smaller than the total with a recorded application site because in several charts the site was documented but the exact dose was not. No statistically significant difference in dose was observed between epifascial and subfascial application (Mann–Whitney U , p=0.285 ).

### 3.2 Serum creatinine levels

**Figure 2:**
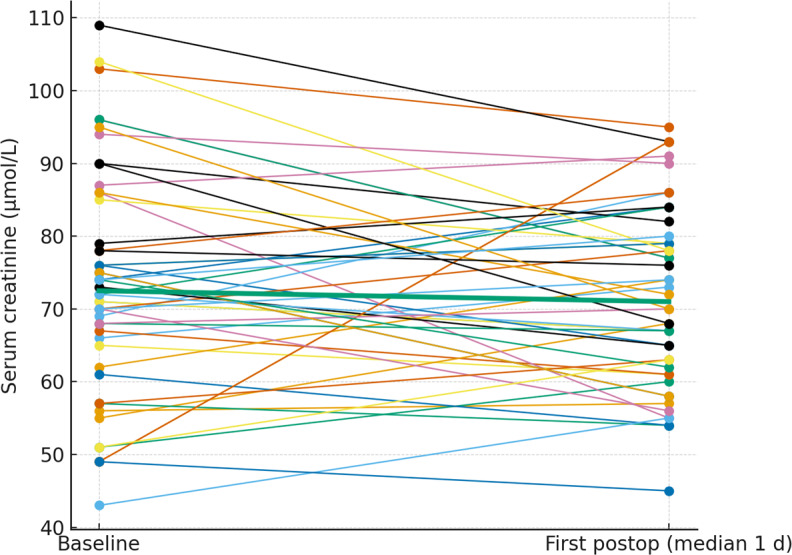
Paired serum creatinine from baseline to the first postoperative measurement. Thin lines depict individual patients; the thick line denotes the cohort median.

**Figure 3:**
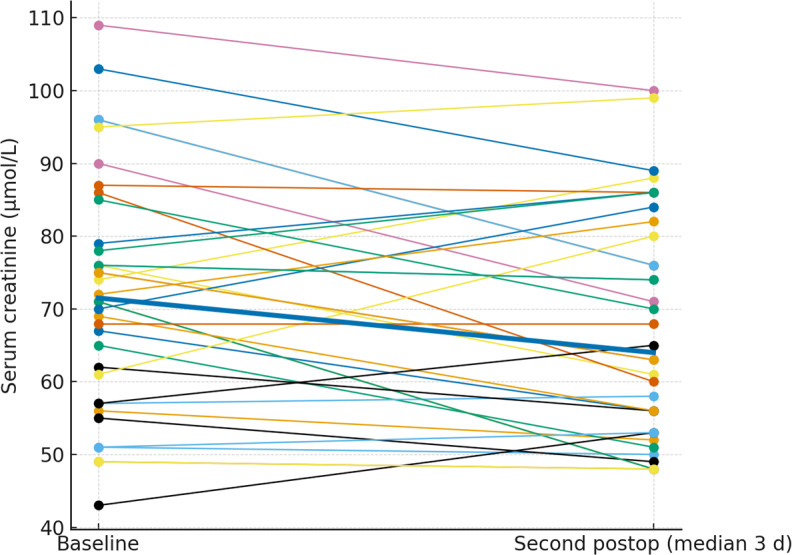
Paired serum creatinine from baseline to the second postoperative measurement. Thin lines depict individual patients; the thick line denotes the cohort median.

Serum creatinine was routinely assessed preoperatively and at two postoperative time points. Only patients with corresponding pre- and postoperative values were included in the analysis. The preoperative measurement served as the baseline reference for comparisons and was included only for patients with corresponding postoperative values. The first postoperative value was collected at a median of 1 d after surgery (mean 1.44 d, SD ± 1.50), while the second was recorded at a median of 3 d (mean 3.56 d, SD ± 1.59). Serum creatinine values were broadly stable at the individual level. The paired plots (Figs. 2 and 3) show patient-level trajectories from baseline to the first and second postoperative measurements without a visible upward shift in the cohort. As the clinically relevant endpoint, acute kidney injury occurred in 2/50 patients, both stage 1; no stage 2 or 3 events were observed. Notably, both AKI events occurred in patients who were exposed to potentially nephrotoxic concomitant medication (discussed below).

### 3.3 Glomerular filtration rate levels

**Figure 4:**
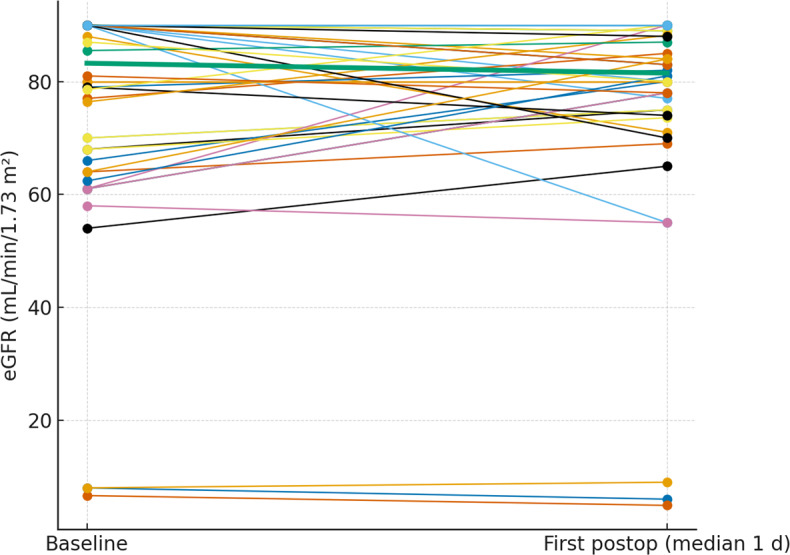
Paired eGFR from baseline to the first postoperative measurement. Thin lines depict individual patients; the thick line denotes the cohort median.

**Figure 5:**
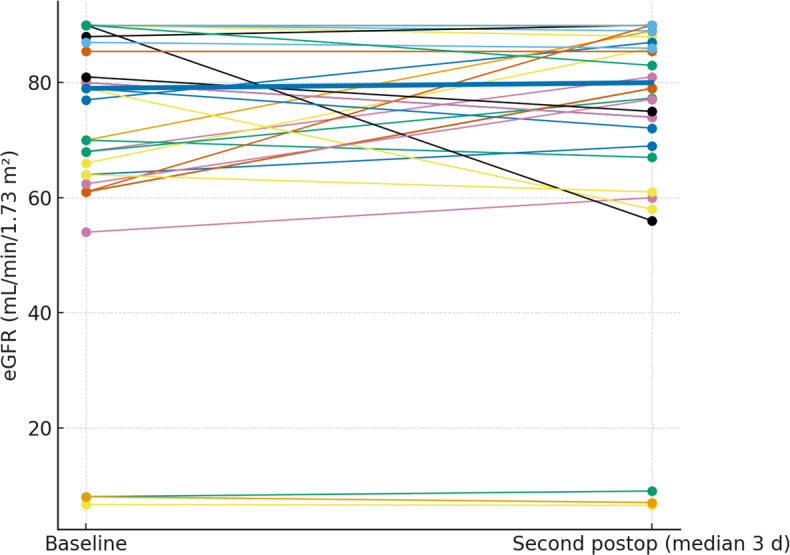
Paired eGFR from baseline to the second postoperative measurement. Thin lines depict individual patients; the thick line denotes the cohort median.

Glomerular filtration rate was assessed at the same time points (median 1 and 3 d). The paired plots (Figs. 4 and 5) show near-flat patient-level trajectories from baseline to the first and second postoperative measurements, with the cohort median essentially unchanged and no systematic downward shift.

### 3.4 Subgroup analysis

We evaluated whether AKI incidence differed across prespecified subgroups: nephrotoxic concomitant medication (yes vs. no), application plane (epifascial vs. subfascial), vancomycin dose ( <1000 vs. ≥1000 mg), and surgical indication (aseptic vs. septic), using Fisher's exact tests. Across all categories, we found no between-group differences in AKI incidence (all p>0.05 ).

## 4 Discussion

This retrospective study aimed to evaluate the renal safety profile of intraoperative local vancomycin powder application across a heterogeneous orthopedic patient population. Our findings demonstrate that neither serum creatinine levels nor estimated glomerular filtration rate changed significantly postoperatively, regardless of vancomycin dosage, application route, surgical indication, or the concomitant use of nephrotoxic medications. These data suggest that the use of intrawound vancomycin powder does not adversely affect short-term renal function and supports its continued use as a safe adjunct for SSI prophylaxis in orthopedic procedures.

In our cohort, even among patients who received doses exceeding 1000 mg or those who underwent subfascial application – presumably associated with higher systemic absorption – no statistically significant renal function changes were observed. While theoretical concerns exist that subfascial application could facilitate greater systemic uptake via exposure to highly vascularized tissues, our findings do not support this hypothesis. The overall AKI incidence was low, and both events were KDIGO stage 1. Notably, both AKIs occurred in patients concurrently exposed to potentially nephrotoxic concomitant medication. While our subgroup analysis did not detect statistically significant between-group differences in AKI incidence, the clustering of events among exposed patients suggests that concomitant nephrotoxins may have contributed to transient creatinine rises rather than an independent effect of topical vancomycin. Given the small sample and low event count, interaction effects cannot be excluded; however, within-patient trajectories of serum creatinine and eGFR showed no systematic postoperative deterioration consistent with a topical vancomycin-related nephrotoxic effect.

The nephrotoxic potential of systemically administered vancomycin is well recognized, with the risk of acute kidney injury varying widely depending on factors such as dosage, treatment duration, and the presence of concomitant nephrotoxic agents. In contrast, local administration mostly bypasses systemic exposure and allows exceptionally high local antibiotic concentrations – often exceeding 1000 × the minimum inhibitory concentration (MIC) for methicillin-resistant *Staphylococcus aureus* – without the risk of systemic accumulation (Martinez-Peralta et al., 2022). This pharmacokinetic profile may explain the low nephrotoxicity signal observed in both the present study and prior investigations.

Despite these reassuring findings, several limitations of our study must be acknowledged. First, the retrospective design introduces a potential for selection and documentation bias. Second, our sample size may limit the detection of rare but clinically significant renal adverse events. Third, due to the lack of serum vancomycin level measurements, systemic absorption can only be inferred indirectly from stable creatinine and eGFR values. Fourth, our analysis was limited to early postoperative changes; we cannot exclude delayed renal effects occurring beyond the observation window. Fifth, the dosing regimens and application planes were not standardized across cases, introducing heterogeneity. Finally, the absence of a control group without vancomycin application limits our ability to distinguish vancomycin-related changes from general perioperative renal fluctuations.

## 5 Conclusion

In this retrospective cohort study, the intraoperative application of local vancomycin powder in orthopedic surgery was not associated with significant postoperative changes in renal function. Across all subgroups, including patients with higher dosages, subfascial application, septic indications, and concomitant nephrotoxic medications, no statistically relevant impairment of serum creatinine or eGFR was observed. These findings support the renal safety of local vancomycin as an adjunctive measure for infection prophylaxis. These findings may reassure surgeons using vancomycin powder that, in routine orthopedic practice, short-term renal risk appears minimal. Nevertheless, well-designed prospective trials with larger sample sizes and standardized pharmacokinetic monitoring are essential to conclusively establish the long-term renal safety of local vancomycin application.

## Data Availability

The underlying dataset was derived from electronic medical records and cannot be shared publicly or on request because of legal and ethical restrictions related to patient confidentiality and institutional data protection policies.

## Code availability

This study did not generate custom software code (e.g., no script-based workflow such as SPSS syntax). Statistical analyses were performed using the proprietary third-party software IBM SPSS Statistics (version 27; IBM Corp.), which is available via a commercial license from the vendor. Therefore, no underlying source code is publicly accessible.
